# Cytotoxic profiling of artesunic and betulinic acids and their synthetic hybrid compound on neurons and gliomas

**DOI:** 10.18632/oncotarget.18390

**Published:** 2017-06-07

**Authors:** Annemarie Ackermann, Aysun Çapcı Karagöz, Ali Ghoochani, Michael Buchfelder, Ilker Eyüpoglu, Svetlana B. Tsogoeva, Nicolai Savaskan

**Affiliations:** ^1^ Translational Cell Biology & Neurooncology Laboratory, Universitätsklinikum Medical School Erlangen, Friedrich-Alexander University of Erlangen – Nürnberg (FAU), Erlangen, Germany; ^2^ Interdisciplinary Center for Molecular Materials (ICMM), Friedrich-Alexander University of Erlangen – Nürnberg (FAU), Erlangen, Germany; ^3^ Department of Neurosurgery, Universitätsklinikum Medical School Erlangen, Friedrich-Alexander University of Erlangen – Nürnberg (FAU), Erlangen, Germany; ^4^ BiMECON Ent., Berlin, Germany

**Keywords:** cell death, cancer cytotoxicity, artesunic acid, betulinic acid, hybrid synthesis

## Abstract

Gliomas are brain-born tumors with devastating impact on their brain microenvironment. Novel approaches employ multiple combinations of chemical compounds in synthetic hybrid molecules to target malignant tumors. Here, we report on the chemical hybridization approach exemplified by artesunic acid (ARTA) and naturally occurring triterpene betulinic acid (BETA). Artemisinin derived semisynthetic compound artesunic acid (ARTA) and naturally occurring triterpene BETA were used to synthetically couple to the hybrid compound termed 212A. We investigated the impact of 212A and its parent compounds on glioma cells, astrocytes and neurons. ARTA and BETA showed cytotoxic effects on glioma cells at micromolar concentrations. ARTA was more effective on rodent glioma cells compared to BETA, whereas BETA exhibited higher toxic effects on human glioma cells compared to ARTA. We investigated these compounds on non-transformed glial cells and neurons as well. Noteworthy, ARTA showed almost no toxic effects on astrocytes and neurons, whereas BETA as well as 212A displayed neurotoxicity at higher concentrations. Hence we compared the efficacy of the hybrid 212A with the combinational treatment of its parent compounds ARTA and BETA. The hybrid 212A was efficient in killing glioma cells compared to single compound treatment strategies. Moreover, ARTA and the hybrid 212A displayed a significant cytotoxic impact on glioma cell migration. Taken together, these results demonstrate that both plant derived compounds ARTA and BETA operate gliomatoxic with minor neurotoxic side effects. Altogether, our proof-of-principle study demonstrates that the chemical hybrid synthesis is a valid approach for generating efficacious anti-cancer drugs out of virtually any given structure. Thus, synthetic hybrid therapeutics emerge as an innovative field for new chemotherapeutic developments with low neurotoxic profile.

## INTRODUCTION

**Figure 1:**
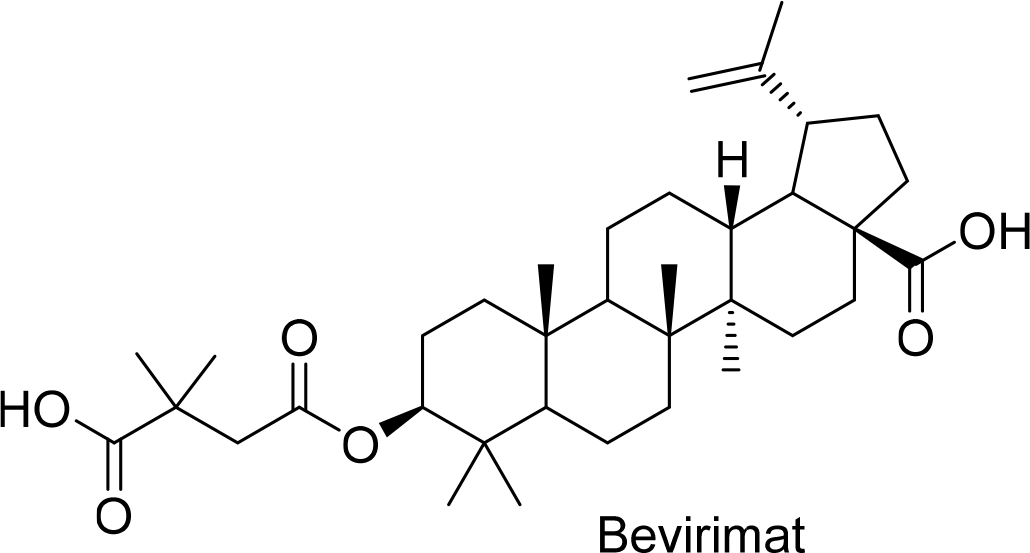
Structure of bevirimat

Primary brain tumors are one of the most threatening conditions in oncology since effective curative therapies still do not exist [1, 2]. The current strike force in treating malignant gliomas (WHO grade III and IV) is it first line surgery aiming at supra-complete tumor removal [2]. A cornerstone in neuro-oncology subsequently to surgery represents chemotherapy with cytotoxic drugs such as temozolomide, vincristin and cisplatin [3, 4, 5] However, cytotoxic strategies have seminal disadvantages such as serious side effects and development of chemo resistance [6, 7]. In fact, current standard chemotherapeutic drugs are mainly cytotoxic and are not selective for targeting cancer cells. Thus, there is an urge for novel compounds with high efficacy, specificity and low unintended side effects. Therefore, developments in cancer therapeutics concentrate on discovering drugs that selectively and effectively operate on cancer cells. Rising attention on natural products and their structures inspires the scientists to use them in drug discovery. During the last 30 years approximately 50 % of the confirmed anticancer drugs are formed from natural products or their structures, such as paclitaxel (Taxol) or campthothecin as important anticancer drugs [8]. Besides, many of the other natural products and their derivatives are under investigations; such as Bevirimat which is a derivative of natural triterpene betulinic acid. Bevirimat [3-O-(3’,3’-dimethylsuccinyl)betulinic acid] is an antiretroviral agent which inhibits viral maturation (Figure 1). Bevirimat shows high activity against viruses *in vitro* and *in vivo.* This promising antiviral compound is in phase IIb clinical trials [9].

**Figure 2:**
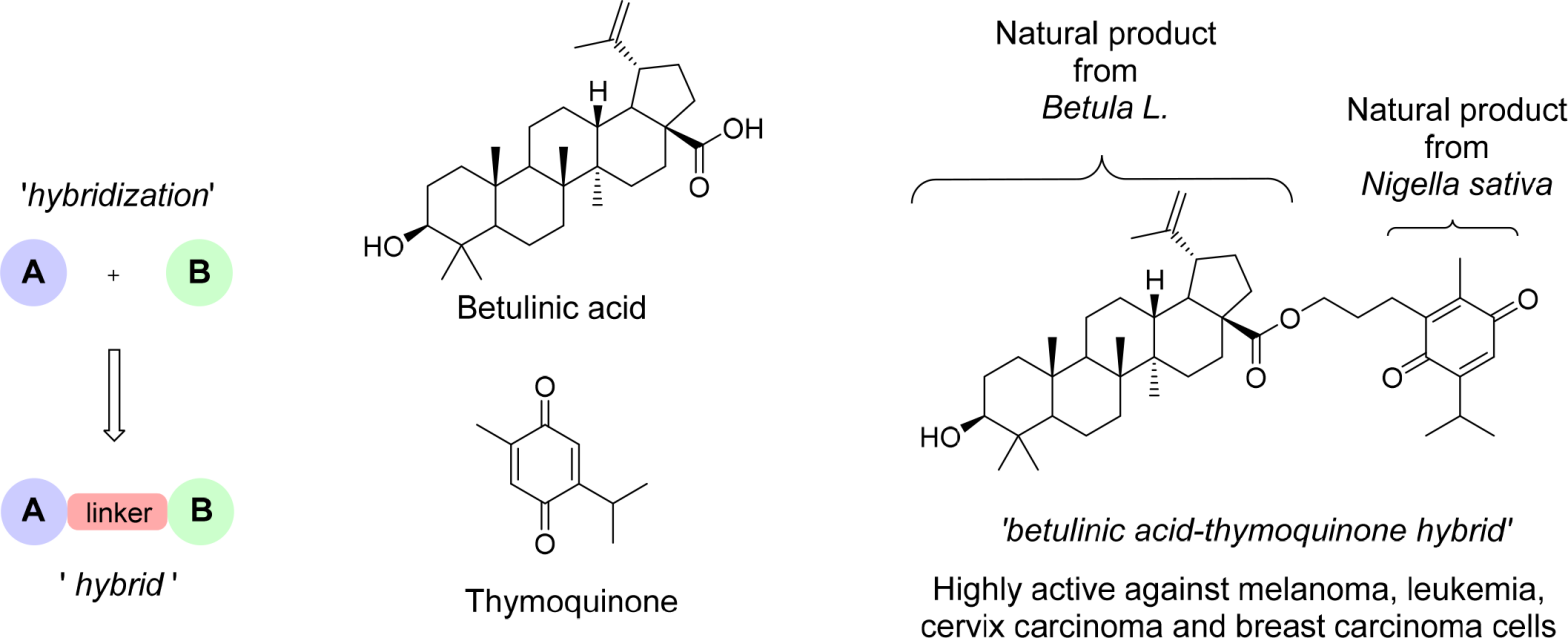
Natural products hybridization Given is a scheme displaying the principle of the chemical hybrid synthesis concept. This chemical hybrid synthesis approach is a valid methodology for generating efficacious anti-cancer drugs out of virtually any given structure. Thus, synthetic hybrid therapeutics emerge as an innovative field for new chemotherapeutic developments.

Another promising and fundamentally novel approach in order to obtain new specific anticancer active compounds with improved pharmacological properties is the hybridization of bioactive natural products: Two or more natural product fragments are combined and linked with each other via covalent bonds forming new hybrid molecules (Figure 2) [10, 11, 12, 13].

These synthetic hybrids containing partial structures of natural compounds are in many cases more active than their parent compounds [14, 15]. As an example, the betulinic acid-thymoquinone hybrid has been reported superior to thymoquinone itself [16]. In the search for new drug candidates that specifically target brain tumors, we focused on the concept of hybridization, encouraged also by our previous results and experiences with artemisinin based hybrids [18, 19, 20, 21]. In this study, we focused on artesunic acid, a water soluble derivative of the natural antimalarial compound artemisinin - an enantiomerically pure sesquiterpene containing a 1,2,4-trioxane ring, which was extracted from the Chinese medicinal plant *Artemisia annua* L. in 1972 by Nobel laureate Youyou Tu [22].

Artesunic acid can induce cell death and oncogenesis in various cancer cells such as in breast cancer cells, T leukemia cells, myeloid leukemia and pancreatic cancer cells [23, 24, 25, 26]. Mechanistically, artesunic acid mediates cytotoxicity via increased reactive oxygen species (ROS) generation. Artesunic acid has been found to induce lysosomal directed cell death, apoptosis, necrosis and ferroptosis dependent of the cell type [23, 26, 27].

As mentioned earlier, another promising class of natural compounds represents betulinic acid (BETA), which is an oxidation product of betulin (with CH_2_OH group instead of COOH at C-28). Particularly BETA itself has been reported as an antitumor agent in many constitutive studies and patents. BETA is a representative molecule from the pentacyclic triterpenoids with proven cell death inducing activity in various cancer cells [28, 29, 30]. Independent lines of research have shown that BETA induces apoptosis in breast cancer cells and melanoma cells [30, 31]. In contrast to ARTA, BETA has been shown to induce cell death also in some glioma cells [32]. Thus, many lines of evidence recognized BETA as a promising candidate as a chemotherapeutic. Strikingly, BETA’s chemical properties such as poor solubility, lipophilicity, and cellular uptake efficacy were the main roadblocks for its routine medical practice [33]. Analogs of this natural product have been synthesized and analyzed to understand its chemistry and biology in order to enhance the properties like hydrosolubility together with higher cytotoxicity. A few of these analogs maintain the high cytotoxicity and selectivity against tumor cells. Attempts to achieve these analogs consist of modifications on the C-3, C-20 and C-28 carbon atoms of BETA structure which might increase the solubility according to previous studies [34].

We followed the strategy to first evaluate the impact of ARTA and BETA on various glioma cells as single compounds and then to perform the combination treatment with a 1:1 mixture of both single drugs. Second, we envisioned the idea of generating a synthetic hybrid of ARTA and BETA in order to combine each molecular properties, thereby boosting the cancer killing potential. Also, we considered the subtoxic and toxic doses on normal cellular constituents of the brain, namely neurons and astrocytes. Emerging evidence exists for enhanced efficacy when compounds are hybridized to increase potency or to generate novel biological functions [18, 19, 20, 21]. Another advantage of hybrid molecules is the inexhaustible variety of potential new moieties with potential higher potency, different pharmacodynamics and specific pharmacokinetics.

Here, we present a comprehensive study on the biological effects of ARTA, BETA, their combinations and the hybrid molecule thereof on malignant brain tumor cells. We found that ARTA exhibits higher cytotoxic potential on rodent gliomas compared to BETA, whereas BETA was more effective on the human cell lines used in this study. Moreover, combined strategies either as hybrid molecules or as combinations of ARTA and BETA even potentiated the biological activities in comparison to single molecule approaches.

## RESULTS

### Generation of synthetic artesunic-betulinic acids hybrid compound

When designing the hybrid molecule, we chose the position C-3 of BETA for coupling so at the position C-28 carboxylic acid group could be free which suggested the cause for higher activity of BETA compared to betulin. Previous studies showed that using various linkers is also affecting the activity, however, in this study the hybrid comprises directly of two compounds without additional linker. The coupling of parent compounds was expected to generate a synergistic effect between the components of the hybrids.

**Figure 3:**
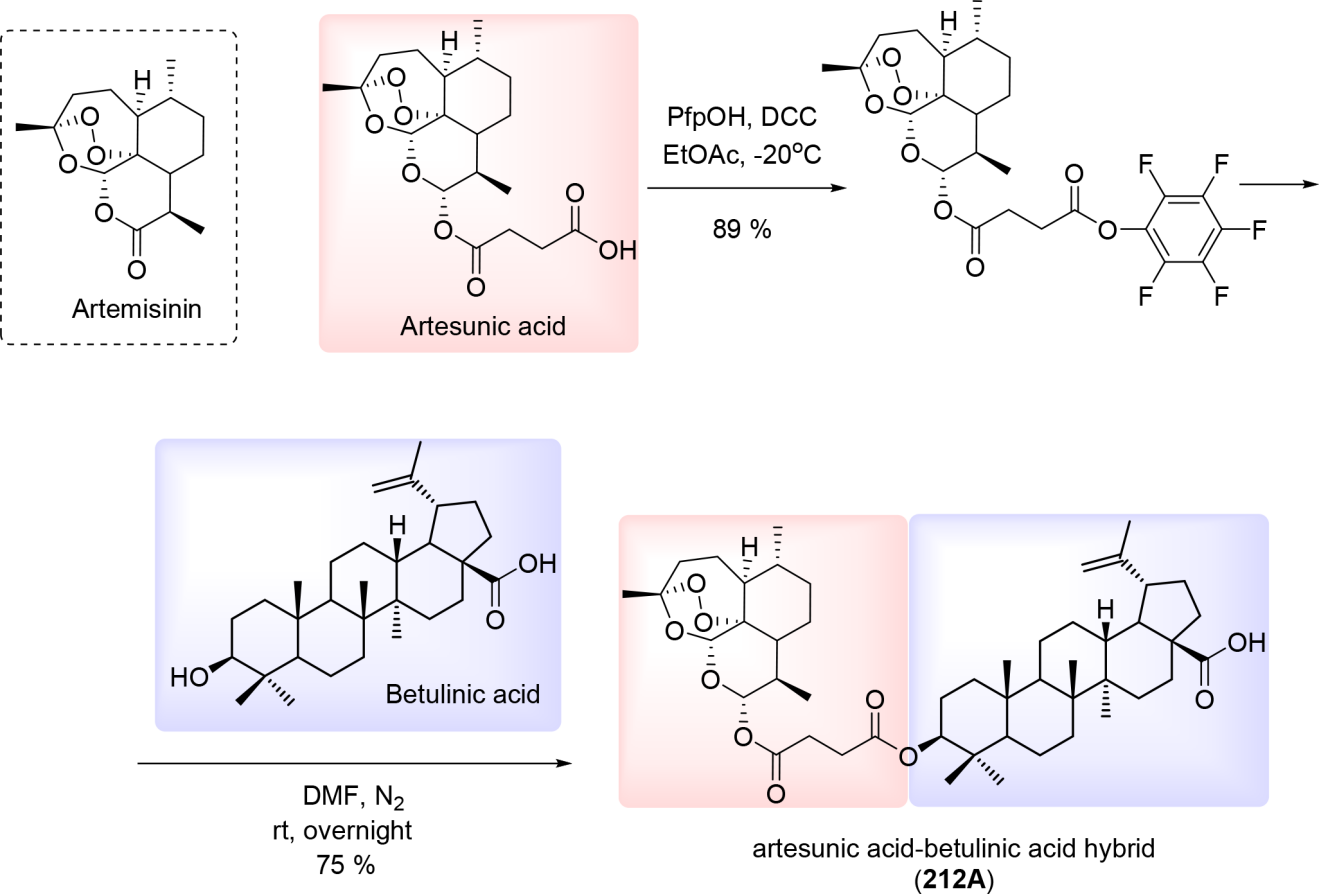
Synthesis of artesunic and betulinic acids hybrid 212A

**Figure 4:**
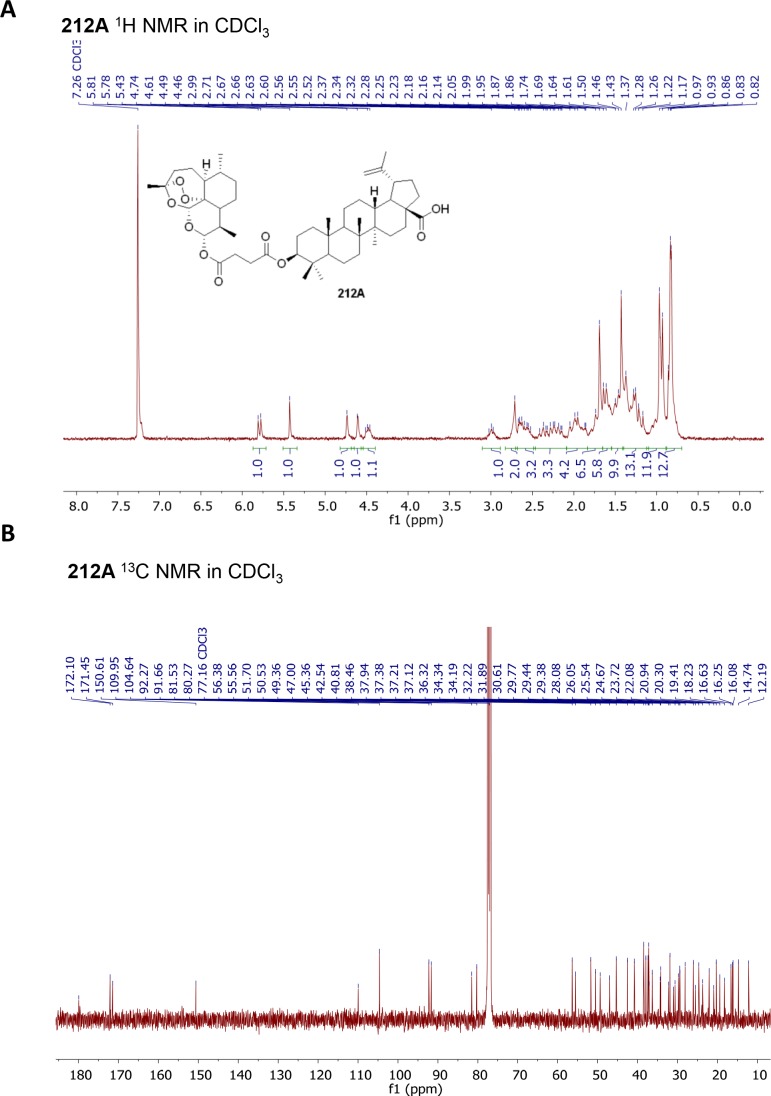

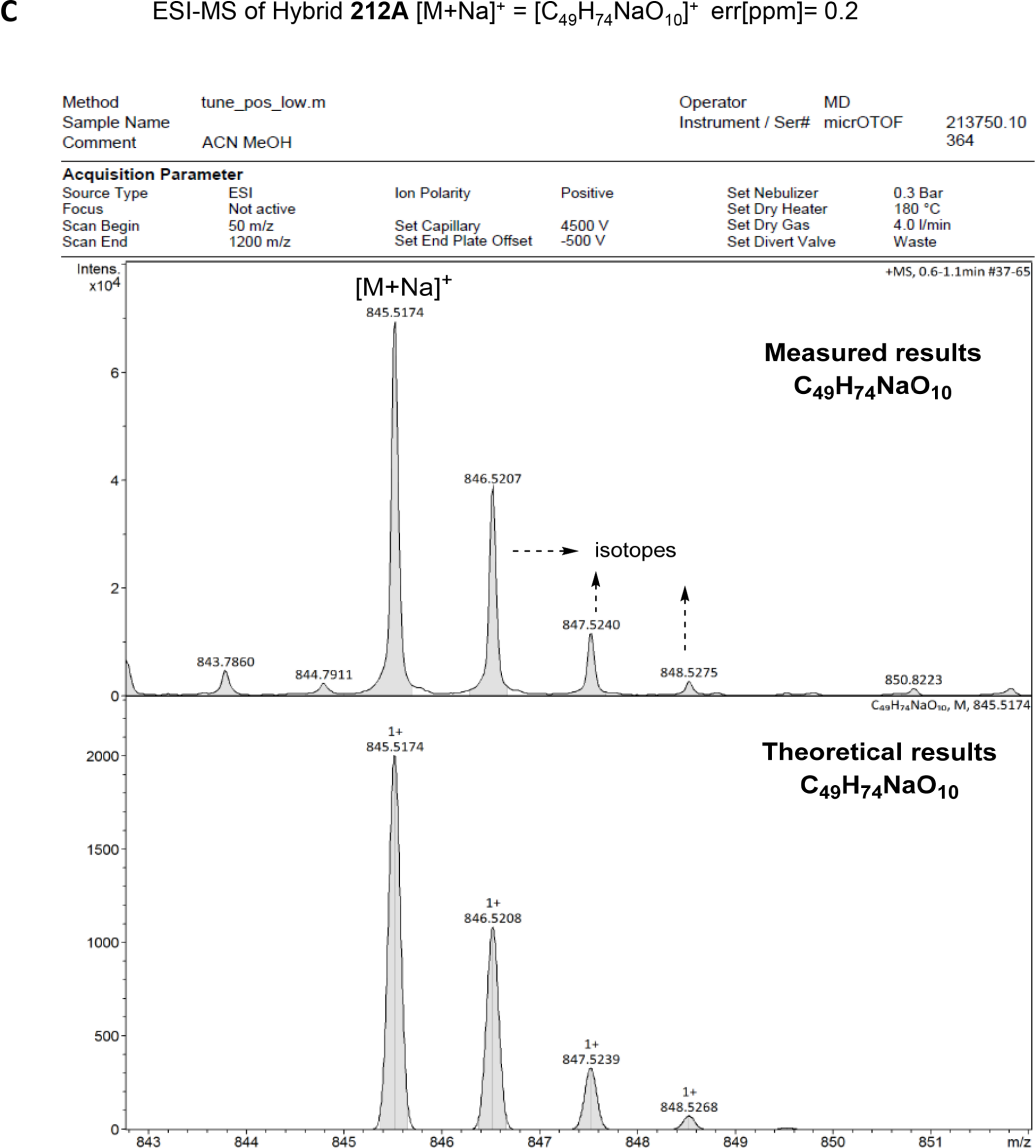

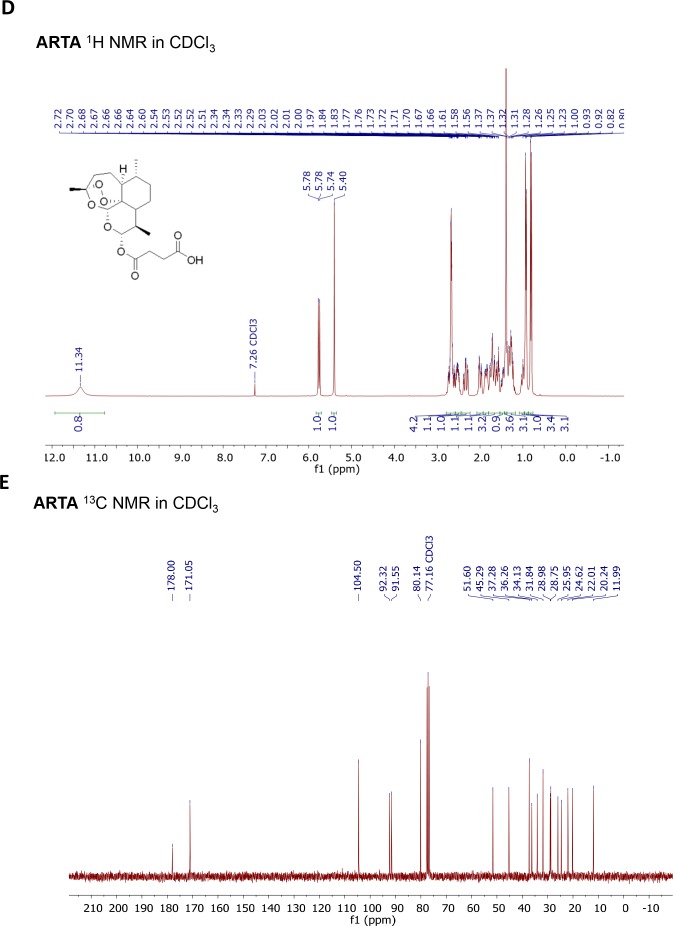

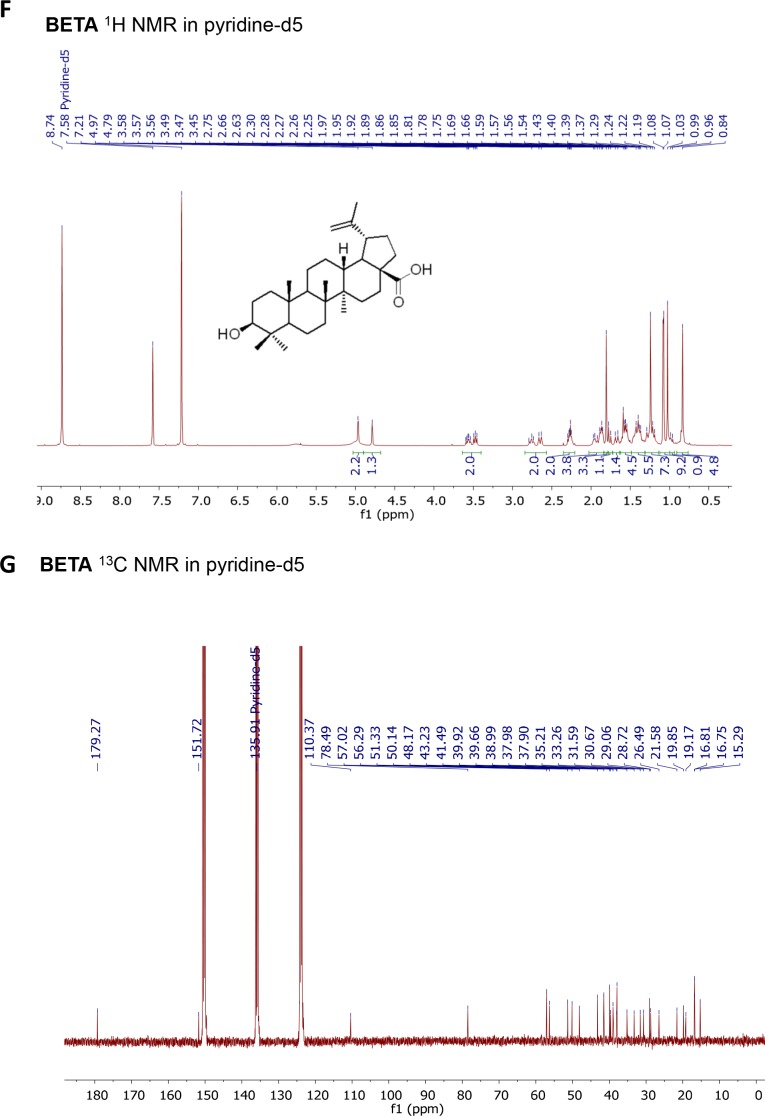
NMR and Mass spectra of hybrid compound 212A, BETA and ARTA (**A)** ^1^H-NMR spectrum (300 MHz, CDCl_3_) of 212A. (**B)** ^13^C-NMR spectrum (75 MHz, CDCl_3_) of 212A. (**C)** ESI-MS spectrum of 212A. (**D)** ^1^H-NMR spectrum (300 MHz, CDCl_3_) of ARTA. **(E)** ^13^C-NMR spectrum (75 MHz, CDCl_3_) of ARTA. (**F)** ^1^H-NMR spectrum (300 MHz, CDCl_3_) of BETA. (**G)** ^13^C-NMR spectrum (75 MHz, CDCl_3_) of BETA.

**Figure 5:**
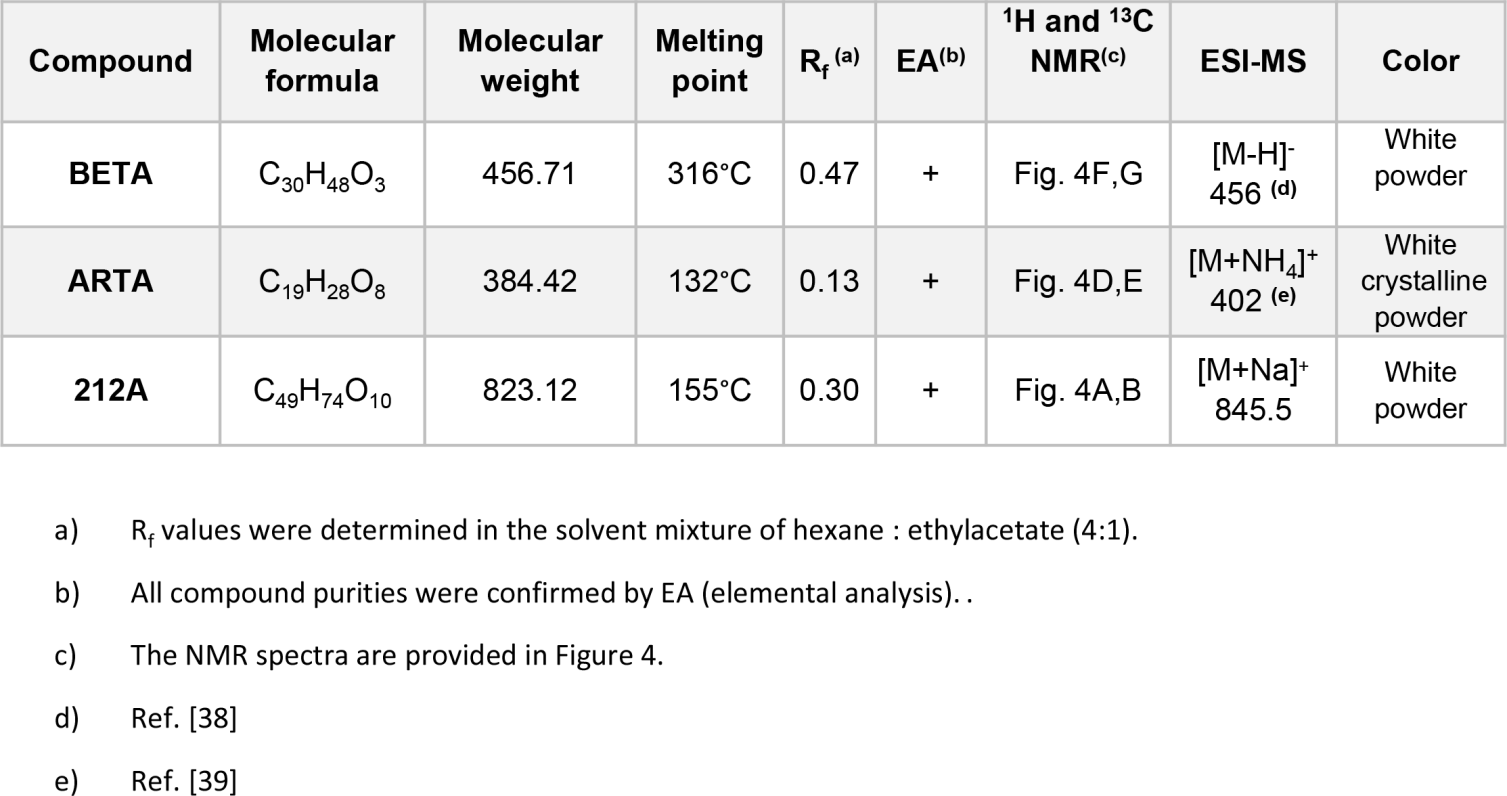
Properties of investigated parent compounds and hybrid 212A R_f_ values were determined in the solvent mixture of hexane : ethylacetate (4:1). All compound purities were confirmed by EA (elemental analysis). The NMR spectra are provided in Figure 4.

First, we synthesized artesunic acid penta-fluorophenyl ester according to established procedures [17]. This active ester was reacted with betulinic acid in dry dimethylformamide (DMF) under N_2_ (Figure 3). The reaction mixture was stirred overnight and the crude product was purified via column chromatography resulting in hybrid 212A with 75 % yield (Figure 3). We further performed ^1^H and ^13^C NMR studies (Figure 4A, 4B) and mass spectra (ESI-MS) analysis for the hybrid compound 212A (Figure 4C, see also Figure 5).

### Artesunic-betulinic acids hybrid induces cell shrinkage cell death in glioma cells

**Figure 6:**
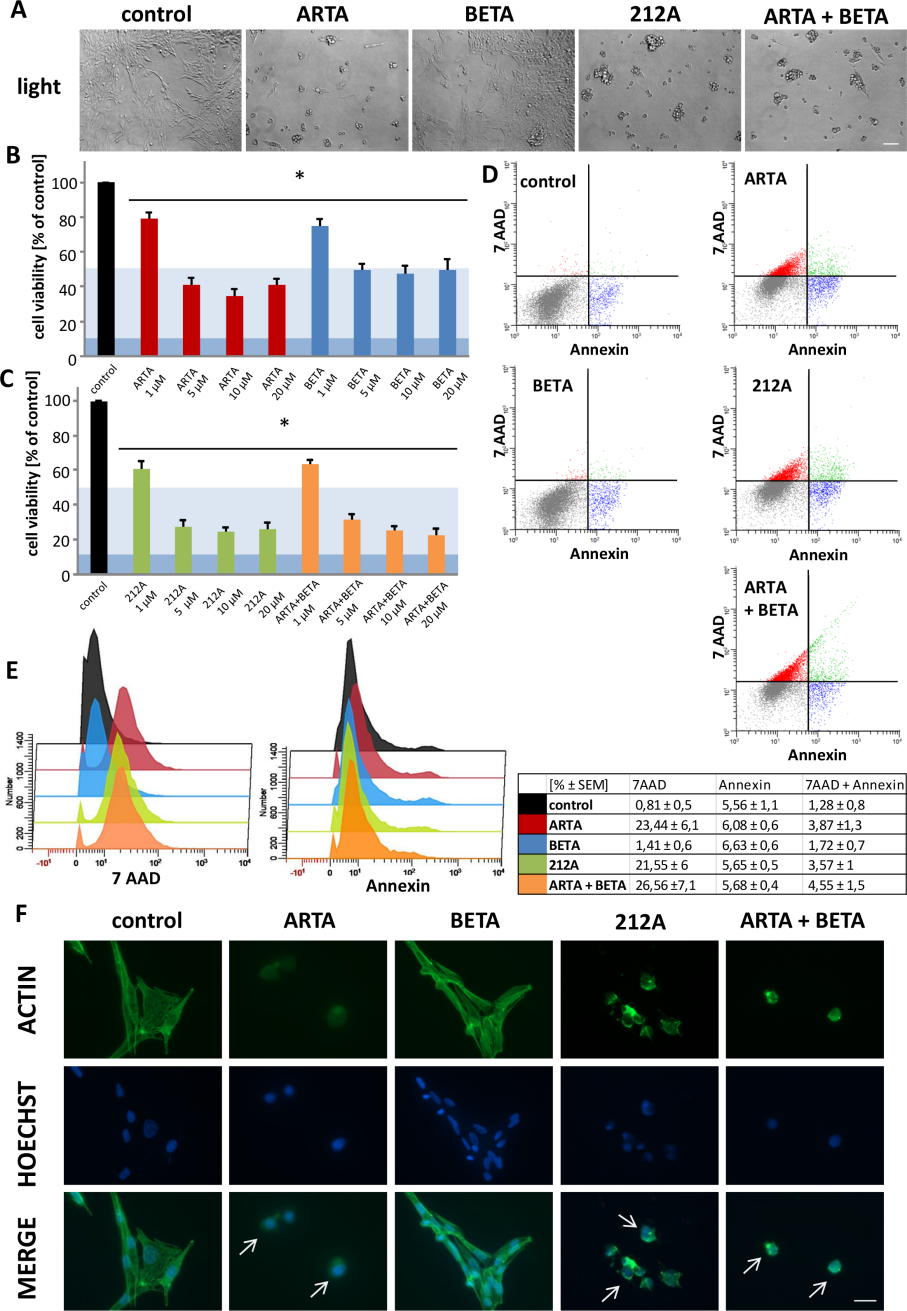
Cytotoxic profile of Artesunic acid, Betulinic acid and the hybrid 212A on rodent glioma cells **(A)** Rat glioma cells (F98) were treated with 5 μM of ARTA, BETA, 212A and ARTA combined with BETA. Cell morphology was examined after 72h with light microscopy. Scale bars, 200 μm. **(B, C)** F98 were treated with different concentrations of ARTA, BETA, 212A and ARTA combined with BETA. Cell viability was measured after 72h. Statistical significance was tested with unpaired two-sided t-test vs. control (n≥ 4; *, p ≤ 0.05). The light blue marking indicates the value of IC_50_ and the dark blue one stands for IC _90_ value. This is passable for the following figures. **(D, E)** Rodent cells were treated with 10 μM of the compounds. Cell death analysis was performed after 24h with 7 AAD and Annexin V. Statistical significance was tested with unpaired two-sided t-test vs. control (n≥ 4; p ≤ 0.05). **(F)** F98 were treated with 10 μM of ARTA, BETA, 212A and ARTA combined with BETA. Cells were fixated and stained with actin marker Phalloidin 488 and DNA marker Hoechst after 48h. Scale bars represent 20 μm.

First, we investigated the effects of artesunic acid, betulinic acid, the combination and the hybrid thereof on glioma cells. Therefore, we facilitated F98 glioma cells which were treated with a wide range of sole artesunic acid (ARTA, for NMR spectra see Figure 4D, 4E), betulinic acid (BETA, for NMR spectra see Figure 4F, 4G) concentrations, their hybrid (212A) and a combination of ARTA and BETA in order to investigate their morphological impact and glioma toxicity potential (Figure 6).

We found, that at the light microscopic level ARTA, 212A and the combination of ARTA and BETA challenged glioma cell morphology dramatically and glioma cells appeared fragmented and shriveled (Figure 6A). In contrast, the effects of BETA on cell morphology were less pronounced (Figure 6A). Our next task was to investigate whether morphological changes match to the effects on the cell viability. In glioma cells, both ARTA and BETA treatment significantly reduced glioma cell viability, although ARTA was more effective (Figure 6B). We found an effective concentration of ARTA at 5 μM to inhibit glioma cell proliferation (Figure 6A, 6B). Furthermore we analyzed the cytotoxic potential of the synthetic hybrid molecule of ARTA and BETA termed 212A and compared the hybrid with the 1:1 combination of the single substances. Combined ARTA and BETA application was more efficient compared to single drug treatment (Figure 6B, 6C). Moreover, the hybrid 212A appeared also highly gliomatoxic although the impact of 212A compared to the combined applications of ARTA and BETA was almost the same (Figure 6C). These findings become even clearer in numbers. The IC_50_ for BETA of 5 μM is the highest, followed by ARTA (IC_50_: 3,8 μM), their combination (IC_50_: 2,3 μM) and 212A (IC_50_: 2 μM) with the lowest concentration needed to achieve a 50 % reduction of cell viability.

Next, we investigated the impact of the compounds on the glioma cytoskeleton. Glioma cells under control conditions showed a polygonal cell shape with many polymerized actin filaments (Figure 6F). Following ARTA application glioma cells presented almost no polymerized actin filaments and nuclei represented a condensed morphology (Figure 6F). In contrast, BETA treated glioma cells displayed only slight morphological changes with reduced cytoplasmatic sizes (Figure 6F). Noteworthy is the effect of 212A on glioma cells. 212A induced massive cell size reduction and increased cortical actin condensation compared to controls (Figure 6F). Also, nuclei showed fainted staining and fragmented morphology (Figure 6F). Combined ARTA and BETA treatment displayed similar morphological effects as 212A showed (Figure 6F).

Altogether, these results demonstrate that all used compounds are effective in reducing glioma proliferation.

### ARTA and 212A are potent gliomatoxic compounds

To analyze whether the reduction in cell viability correlates with gliomatoxicity or might have other reasons we performed a flow cytometry with the marker 7-Amino-actinomycin (7-AAD) and Annexin V. Therefore, cells were treated with 10 μM of the compounds for 24h (Figure 6D, 6E). The results revealed a remarkable significant induction of 7-AAD positive cells which were treated with ARTA, 212A and the combination of ARTA and BETA (Figure 6D, 6E). This shows a major increase in cell death of any kinds of the cells treated with one of these substances. However, treatment of cells with BETA alone showed no considerable difference compared to the control group in terms of dead cells (Figure 6D, 6E). We next investigated Annexin V staining following treatment. Interestingly, all tested compounds revealed almost the same amount of Annexin V positive cells (Figure 6D, 6E). These data indicate that 212A and its parent compounds do not differ in inducing early apoptosis. A small part of the examined cells in each compounds group were both positive for Annexin V and 7-AAD (Figure 6D, 6E). Controls and cells treated with BETA showed with around 1,5% the lowest amount of cells positive for both markers (Figure 6D, 6E). In contrast, ARTA, the hybrid and combination of ARTA and BETA displayed a higher number of stained cells for either dye on a comparable high level compared to controls (Figure 6D, 6E). This leads to the conclusion that only a small part of the cell death induced by the ARTA-related compound groups and ARTA itself is caused via apoptosis.

In summary, this experiment shows that the decrease in the metabolic activity and change of morphology of the cells treated with ARTA, 212A and the combinatorial treatment with ARTA and BETA can be attributed to a high induction of cell death. Whereas the results of the cells treated with BETA indicate that though we showed in our previous assays that the metabolic activity is decreased by this compounds, the cells might still be alive. Regarding the mechanism of cell death, the results suggest that the major part of the cells treated with ARTA or one of the ARTA related substances did not die via apoptosis because most of the cells were solely positive for Annexin V.

### Artesunic acid, betulinic acid and their hybrid 212A show toxic effects on human glioma cells

Next, we examined the impact of our substances on human glioma cells. Therefore, we treated U87 and TN22 with ARTA, BETA, 212A and the combination of the single compounds with different concentrations (1 μM, 5 μM, 10 μM and 20 μM) for again 72h.

**Figure 7:**
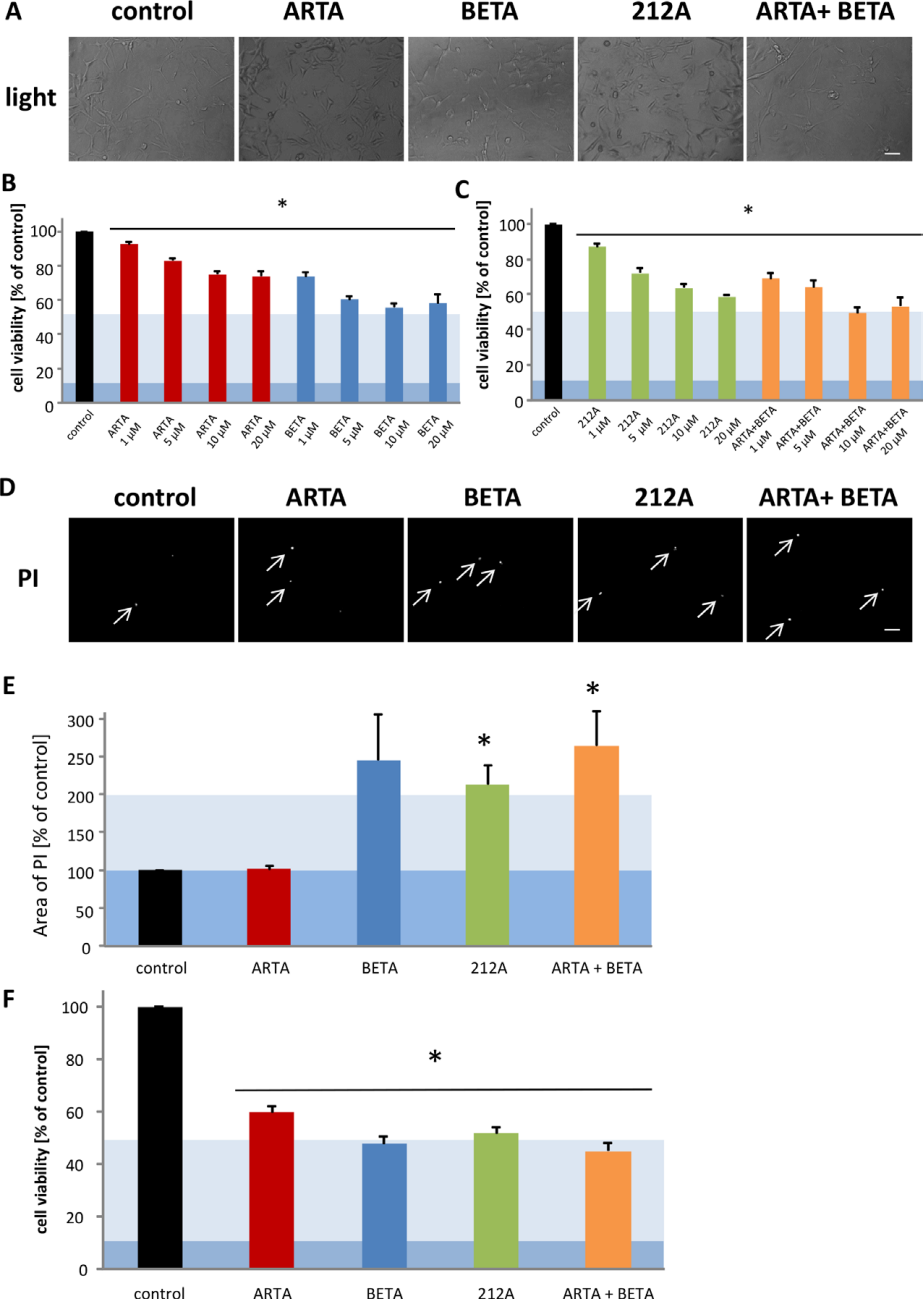
Cytotoxic effects of Artesunic acid, Betulinic acid and the hybrid 212A on human glioma cells **(A, D)** U87 MG (human glioblastoma/astrocytoma cells) were treated with 10 μM of ARTA, BETA, 212A and ARTA combined with BETA. After 72h representative images with propidium iodide (PI) and light microscopy were taken. Scale bars, 200 μm. **(B, C)** U87 were treated with different concentration of ARTA, BETA, 212A and ARTA combined with BETA. Cell viability was measured after 72h. Statistical significance was tested with unpaired two-sided t-test vs. control (n≥3; *, p ≤ 0.05). **(E)** Cells were treated with 10 μM of ARTA, BETA, 212A and the combinatorial treatment of ARTA and BETA. After 72h, cell death was examined with propidium iodide (PI) staining. The images were quantified and related to the control. Statistical significance was tested with unpaired two-sided t-test vs. control (n≥4; *, p ≤ 0.05). (**F)** TN22 (human glioma cells) were treated with 10 μM of ARTA, BETA, 212A and ARTA combined with BETA. Cell viability was measured after 72h. Statistical significance was tested with unpaired two-sided t-test vs. control (n≥4; *, p ≤ 0.05).

Interestingly, none of the compounds or combinations showed a significant effect on the morphology of the human glioma cells in light microscopy (Figure 7A). The next step was to examine the cell viability. All used treatments reduced the cell viability significantly. Noteworthy, ARTA had the least impact on the cells, already the treatment with 1 μM with BETA showed a similar effect as 20 μM of ARTA (Figure 7B). Whereas the effect of BETA and the combinatorial treatment with ARTA were most effective especially in lower concentrations (Figure 7B, 7C). Comparing the reduction in cell viability after the application of 212A or ARTA in combination with BETA, our experiments disclosed that in higher concentration (20 μM) both decreased the cell viability by about the same level, while the combination of the single compounds seemed more effective in lower concentrations significantly even if using only 1 μM (Figure 7C). Further, we analyzed whether our findings of the cell viability assay can be also seen performing a propidium iodide staining. The propidium iodide staining indicated a higher rate of cell death in the groups treated with BETA, 212A and the combination of ARTA and BETA compared to control (Figure 7D, 7E). We found the highest amount of cell death at the combination treatment with ARTA and BETA and the single treatment of BETA (Figure 7D, 7E). ARTA did not show any effect compared to the cells without treatment regarding the propidium iodide staining (Figure 7D, 7E). Since these findings match to our previous experiments, it seems that the reduction of cell viability is caused mostly by cell death. To support the results so far made, we measured the cell viability of another human glioma cell line (TN22) for one concentration (10 μM) as an example. Also when treating TN22 BETA, its combination with ARTA and 212A showed the greatest impact on the glioma cells (Figure 7F).

Comparing all these findings with the results of the rodent cell line it can be noticed that, whereas ARTA seemed more effective when treating F98 (Figure 6B), BETA showed a significant greater effect in reducing the cell viability compared to ARTA on the human cell line U87 and TN22 at lower concentrations (1μM, 5μM, 10μM) (Figure 7B). Furthermore in these experiments we showed that all substances decreased the cell viability of human glioma cells and that especially for BETA, 212A and the combination of ARTA and BETA this can be attributed to an increase of cell death (Figure 6B-6E). This indicates that though the cell death analysis we performed with the rodent cell line showed that the reduction of cell viability of the cells treated with BETA might not be caused by cell death, BETA can still be seen as a potential gliomatoxic drug for human cells.

### Artesunic acid and betulinic acid show no significant neurotoxic and gliotoxic impact at low concentrations

**Figure 8:**
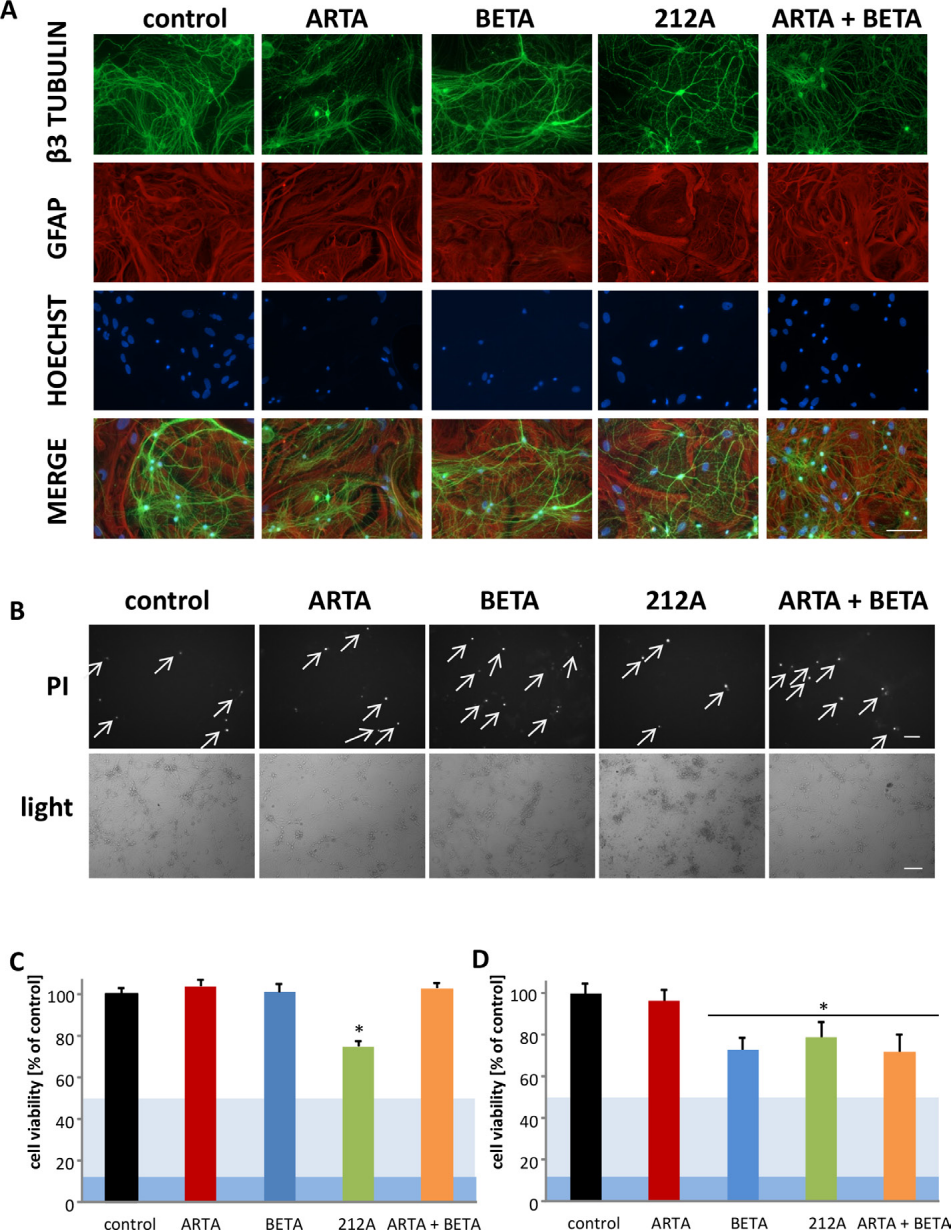
Neurotoxicity and gliomatoxicity profiling of Artesunic acid, Betulinic acid and the hybrid 212A **(A)** Primary neurons and astrocytes were treated with 10 μM ARTA, BETA, 212A and ARTA in combination with BETA. After 72h cells were fixed and immunostained for the neuronal marker beta-III- tubulin, the glial marker GFAP and the DNA marker Hoechst. Scale bars, 100 μm. **(B)** Primary neurons and astrocytes were treated with 10 μM of ARTA, BETA, 212A and ARTA combined with BETA. Cell death was examined after 72h with propidium iodide (PI) and light microscopy. Scale bars, 200 μm. **(C, D)** Primary neurons and astrocytes were treated with 5 μM **(C)** and 10 μM **(D)** of ARTA, BETA, 212A and ARTA combined with BETA. Cell viability was measured after 72h. Statistical significance was tested with unpaired two-sided t-test vs. control (n≥5; *, p ≤ 0.05).

The next step was to investigate the ARTA compounds and BETA for their impact on healthy brain cells. Therefore, primary neurons and astrocytes were extracted from rodent brain and treated with 5μM and 10μM of ARTA, BETA, their combination and the hybrid 212A (Figure 8). First, to examine the morphological effects we fixed the treated cells and analyzed the cytoskeleton. There were no changes visible in structure or density between the treated groups and controls. Neurons showed complex dendritic arborization which was not affected by ARTA, BETA or 212A (Figure 8A). To investigate the number of dead cells we stained neuronal cells co-cultured with astrocytes with propidium iodide staining after treatment with a concentration of 10 μM for each compound with duration of 72h. An increased number of cell death was displayed following treatment with BETA and the combination of ARTA with BETA (Figure 8B). These results correlate partially with our findings of the cell viability assays after treating cells with a concentration of 10 μM, whereas ARTA showed no significant difference compared to controls. In the other three treatment groups (containing BETA, 212A and the combination of ARTA and BETA) a decrease in cell viability was detected (Figure 8D).

Next step was to find out how primary neurons and astrocytes react, exposed to a lower concentration of these compounds. For this we applied a concentration of 5μM for each compound and their combinations. The cell viability assays indicated no significant neurotoxic and gliotoxic effect for ARTA, BETA and their combination, whereas 212A still had a significant impact (Figure 8C).

Overall these results combined with the toxic effect on the glioma cells already at the concentration of 5 μM demonstrate the value of ARTA and BETA to combat gliomas, especially ARTA, with no change in cell viability at higher concentrations (10 μM).

### Artesunic acid, the combination of artesunic and betulinic acids and their hybrid 212A inhibit the migration of glioma cells

**Figure 9:**
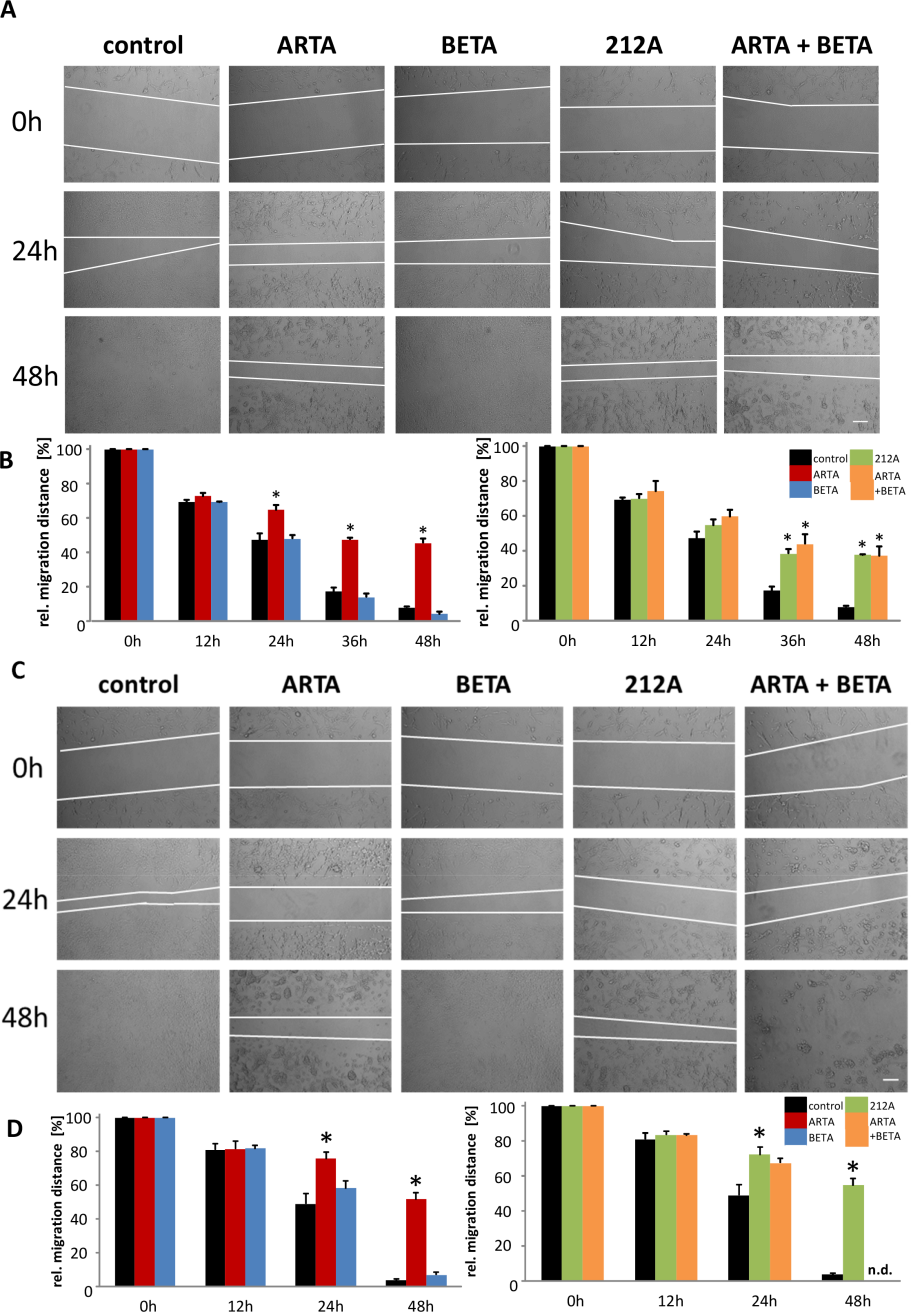
Extent of inhibition of cell migration by Artesunic acid, Betulinic acid and the hybrid on rodent glioma cells **(A, C)** A scratch was made into plated cells which were then treated with 5 μM **(A)** and 10 μM **(C)**. Results were observed at different times. **(B, D)** Migration distance of the treatment with 5 μM **(B)** and 10 μM **(D)** were quantified and compared to the data from 0 h. Statistical significance was tested with unpaired two-sided t-test vs. control (n=3; *, p ≤ 0.05).

Next, we investigated the impact of ARTA, BETA and 212A on glioma migration since this biological feature is clinically highly relevant and has not been solved up to now. Therefore, cells were plated into a 12-well-plate, after 36h a scratch was made, afterwards cells were treated with 5 μM (Figure 9A, 9B) and 10 μM (Figure 9C, 9D) of the compounds and monitored at various times. 12h after the treatment there is no difference in migration visible between the treated cells and the control in both concentrations (Figure 9). Whereas after 24h, we observed a significant difference of the migration distance between the control and artesunic acid in both the lower and the higher concentration, though betulinic acid showed no or only a slight difference in migration compared to the control after 24h (Figure 9B, 9D). Also cells treated with 212A with the higher concentration showed significantly less migration, whereas the decrease in migration in cells treated with the combination of single ARTA and BETA was less pronounced (Figure 9D). After 36h, besides the ARTA and 212A treatment, the experiment also showed a significant decrease in migration for the combined ARTA and BETA treatment (Figure 9A, 9B). After 2 days all treatment groups including ARTA (ARTA, 212A, ARTA + BETA) showed significant less glioma cell migration (Figure 9). The cells treated with 10 μM of ARTA combined with BETA were less attached to the plate and settled in groups so that these results could not be quantified (Figure 9C). In contrast, BETA did not show any effects on glioma cell migration (Figure 9). Interestingly comparing the migration distance between the concentrations there is a significant difference between cells treated with 5 μM and 10 μM of 212A at 24h and 48h though this phenomenon cannot be identified for sole ARTA, BETA and their combination treatment (Figure 9B, 9C).

In summary, these results show that ARTA, 212A and the combination of ARTA and BETA inhibit glioma cell migration on more or less the same level, whereas no such effect was found for BETA.

## DISCUSSION

Still, malignant gliomas are primary brain tumors with fatal outcome. The frontline therapy in most glioblastoma patients with or without neurosurgery represents chemotherapy [4]. Chemotherapy aims at killing glioma cells at concentrations where normal brain cells have chances to survive. This cytotoxic strategy therefore depends on compounds which fulfill the requirements to be specifically toxic to cancer cells. Thus, major focus is on discovering agents or small molecules with high ability to passively diffuse across the blood-brain barrier and impact specifically on glioma cells [2]. We describe here a new therapeutic technology to combat gliomas by combining small molecules through the chemical hybridization approach [10]. This concept itself is not novel; however, the application of such hybrid molecules in neuro-oncological settings and in particular in glioma research has to our knowledge not been described before.

We found a cytotoxic effect of BETA, already using concentrations of 5 μM which correlates with previous studies on malignant brain-tumor cells [29]. In fact, betulinic acid (BETA) is a promising anti-cancer natural compound and a representative molecule from the pentacyclic triterpenoids. BETA has been shown to be toxic in various cancer cells such as in breast cancer cells and melanoma cells [28, 30, 31]. Thus, many lines of evidence recognized BETA as a promising chemotherapeutic candidate. Strikingly, BETA’s chemical properties such as poor solubility, lipophilicity, and cellular uptake efficacy were the main roadblocks for its routine medical practice [33] which may explain why BETA has only some pre-clinical studies [35].

In contrast, artesunic acid entered clinical medicine with the application in malaria infection [22]. ARTA is a water-soluble derivative of artemisinin which was extracted from the Chinese medicinal plant *Artemisia annua* L. ARTA can induce cell death and oncogenesis in various cancer cells such as in breast cancer cells, T leukemia cells, myeloid leukemia and pancreatic cancer cells [23, 24, 25, 26]. Mechanistically, ARTA mediates cytotoxicity via increased reactive oxygen species (ROS) generation lysosomal directed cell death, apoptosis, necrosis and ferroptosis dependent of the cell type [23, 26, 27]. However, somehow unexpected was the efficacy of ARTA on gliomas. ARTA appears gliomatoxic at low concentrations and at the same time exhibits only minor toxic effects on primary neurons and astrocytes. This motivated us to test ARTA and ARTA derived hybrid combined with the known compound BETA. In fact, previous work of our laboratory and others revealed promising effects of ARTA derived hybrids against *Plasmodium falciparum*, multidrug-resistant leukemia cells and viruses [18, 19, 20, 21].

We set out the strategy to investigate the impact of ARTA and BETA on various glioma cells as single compounds, in combination with a ratio of 1:1 mixture of both single drugs and as a hybrid of ARTA and BETA. The hybrid approach was aimed to combine the advantage of both compounds to boost the cancer killing potential. In fact, 212A revealed the lowest IC_50_ value in rodent gliomas. Another feature of 212A is that glioma cell migration can be mitigated by the hybrid 212A. This is an interesting finding, since BETA has almost no effects on glioma migration. However, the differences in glioma toxicity between ARTA and 212A are low. In addition, 212A has a certain neurotoxic potential at least at high levels. Since ARTA shows almost no unintended side effects it is unlikely that 212A will be a realistic candidate for clinical applications in neuro-oncology. Although there is a quest for new small molecule compounds which should turn into novel therapies, our study also shows that at least in the case of ARTA and BETA, combined treatments are equally efficient as hybrids. In this work, artesunic acid and betulinic acid were investigated for the first time against glioblastoma. This is the main focus of the present work. In addition, combinational experiments have been performed, and one synthetic hybrid of both acids has been prepared and investigated. The motivation to covalently bind artesunic acid and betulinic acid can be explained by our previous excellent experiences with hybridization of parent compounds resulting in highly active lead structures. However, the newly synthesized ARTA-BETA hybrid is not outperforming its parent compounds. Nonetheless, variation of linkers might result in highly active hybrid molecules, since the type and the length of the linker play a crucial role for the biological activity of a hybrid drug. However, this is out of scope of the current study.

Altogether, our data reveal that the synthetic hybrid approach can enhance chemotherapeutic potential and helps in optimizing molecular features of therapy. The featured synthetic hybrid concept opens up the possibility of combination of virtually any molecular structure and thus unlimited variations.

## MATERIALS AND METHODS

### Chemicals and general information

Artesunic Acid (ARTA) and DMF were purchased from ABCR (Karlsruhe, Germany) and Sigma Aldrich (Taufkirchen, Germany) (95 % pure). Betulinic Acid (BETA) was purchased from ABCR (Karlsruhe, Germany) (90 % pure), additionally this compound was purified via column chromatography to achieve >98 % purity (Figure 5).

Thin layer chromatography (TLC) was performed on pre-coated aluminum sheets ALUGRAM^®^ SIL G/UV254 (0.2 mm silica gel with fluorescent indicator, MachereyNagel & Co). ^1^H-NMR (^13^C-NMR) spectra were recorded at room temperature on a Bruker Avance spectrometer operating at 300 MHz. All chemical shifts are given in the ppm-scale and refer to the nondeuterized proportion of the solvent. ESI mass spectrum was recorded on a Bruker Daltonik micrOTOF II focus TOF MS-spectrometer. Elemental Analysis (C, H, N), carried out with an Elementar vario MICRO cube machine, is within ±0.40% of the calculated values confirming a purity of >95%.

For our biological studies all drugs were solved under sterile conditions in dimethylsulfoxide (DMSO) to concentration of 20mM.

### Synthesis of pentafluorophenyl ester of artesunic acid

The solution of N,N′-dicyclohexylcarbodiimide in dry ethylacetate was added to a stirred solution of artesunic acid and pentafluorophenol in dry chloroform at -20°C under nitrogen (N_2_) atmosphere. The reaction mixture was stirred overnight at 0°C. The precipitated urea was filtered and the solvent evaporated under vacuum. Afterwards, the crude was purified by column chromatography to give 89% of yield (Figure 3).

### Synthesis and characterization of 212A

In a flame-dried flask under a nitrogen atmosphere, betulinic acid (150 mg, 0.33 mmol, 1.0 equiv.) was dissolved in anhydrous dimethylformamide (DMF) (1 mL) under nitrogen (N_2_) atmosphere. Artesunic acid pentafluorophenyl ester (181 mg, 0.33 mmol, 1.0 equiv.), synthesized according to published procedure [17], was added to the solution of betulinic acid in anhydrous DMF under N_2_ atmosphere. The reaction mixture was stirred overnight. After removing the solvent under reduced pressure the obtained crude product was purified by column chromatography using the solvent mixture hexane/ethyl acetate, Rf = 0.3 ( hexane: ethylacetate 4:1 and indicated with phosphomolybdic acid) as eluent to yield hybrid 212A with 75% yield as white powder (Figure 3 and Figure 5). Melting Point: 154.6 °C. ^1^H NMR (300 MHz, CDCl_3_): δ= 0.74 (m,12H), 0.86 (m, 10H), 1.15 (m, 15H), 1,41 (s, 7H), 1.53 (m, 8H), 1.67 (s, 4H), 1.84 (m, 4H), 2.17 (m, 3H), 2.53 (m, 3H), 2.69 (s, 2H), 2.97 (m, 1H), 4,44 (m, 1H), 4.59 (s, 1H), 4.71 (s, 1H), 5.41 (s, 1H), 5.76 (d, 1H) ppm. ^13^C NMR (75 MHz, CDCl_3_): δ= 12.10, 14.65, 15.99, 16.16, 16.53, 18.13, 19.32, 20.20, 20.84, 21.98, 23.62, 24.56, 25.44, 25.94, 27.98, 29.28, 29.33, 29.67, 30.50, 30.92, 31.78, 32.12, 34.09, 34.22, 36.21, 37.00, 37.09, 37.26, 37.83, 38.34, 40.69, 42.42, 45.23, 46.87, 49.23, 50.40, 51.57, 55.42, 56.23, 80.10, 81.36, 91.48, 92.09, 104.44, 109.74, 150.35, 171.17, 171.81, 179.65 ppm. ESI-MS [M+Na]^+^: calculated for [C_49_H_74_NaO_10_]^+^: 845.5180, found 845.5174. Purity of 212A was confirmed using elemental analysis. Anal. Calcd for C_49_H_74_O_10_: C, 71.50; H, 9.06; Found: C, 70.98; H, 9.06.

### Cell culture glial cell lines

Glioma cell lines (F98, U87) were obtained from ATCC/LGC-2397 (Germany). TN22 cells were generated from surgical resections of glioblastoma patients after informed consent. Cells were cultured under standard humidified conditions (37°C, 5 % CO_2_) with Dulbecco’s Modified Eagle Medium (DMEM; Biochrom, Berlin, Germany) supplemented with 10 % fetale bovine serum (Biochrom, Berlin, Germany), 1 % Penicillin/Streptomycin (Biochrom, Berlin, Germany) and 1 % Glutamax (Gibco/Invitrogen, California, USA). Cells were passaged at approx. 90 % confluence. For passaging cells were washed with PBS and trypsinized. After centrifugation (900 xT, 5 min, 24 °C) cells were plated into culture flask.

### Cell culture primary neurons and astrocytes

For hippocampal neuronal cultures one to four days old Wistar rats (Charles River, USA) were sacrificed. After removing the hippocampi from the brain they were transferred into ice cold Hank’s salt solution. The dentate gyrus was cut away. The so obtained brain tissue was trypsinized (5mg/ml), triturated mechanically and plated into 12-well-plates with MEM medium, supplemented with 10 % fetal calf serum and 2% B27 Supplement (all from Invitrogen, Taufkirchen). After a short time period the medium was replaced with Neurobasal A (Invitrogen, Taufkirchen) and neurons were kept until full differentiation.

### Cell proliferation analysis and toxicity assay

The cell viability assay was performed using 3(4,5 dimethylthiazol) - 2,5 diphenyltetra-zolium (MTT) assay according to Hatipoglu et al. [36]. 3000 cells/ well were plated in 96-well plates, the drug treatment followed two hours later. After 72 hours cells were incubated with MTT solution (Roth, Karlsruhe, Germany) (5 mg/ml) for 4 h at 37°C, 5% CO_2_. The following lysis of the cells occurred with 100 μl isopropanol + HCl (110 ml Isopropanol + 330 μl HCl) for 30 minutes. The mentioned setting was used for the cells F98, U87 and TN22. Primary neurons and astrocytes were seeded into 12-well plates and treated at day 44. After 72h cells were incubated with MTT solution as described earlier. After 4h cell lysis was performed with 200 μl isopropanol + HCL. 90 μl of this solution was transferred into 96-well plate for measuring with the microplate reader. The optical density of each well was measured using the microplate reader Tecan Infinite F50 (Crailsheim, Germany) set to 550 nm (wavelength correction set to 690 nm) using i-control software. Cells without drug treatment were used as control. The viability of the different drug treatments is expressed as the percentage of control.

### Cell death assay and apoptosis analysis

For the cell death assay cells were plated and treated the same way as the MTT assay. Cells were incubated with propidium iodide staining (PI) (Invitrogen, Darmstadt, Germany) for 20 min [1 μg/ml]. Images were taken with Olympus x71 and cell-F-Software (Olympus, Tokyo, Japan). For further analysis 200.000 cells/well were seeded into 6-well-plate and treated after 2h with 10 μM. After 24h medium was collected and cells were trypsinized. Cells were washed with PBS and stained with 0,1% Annexin V and 0,1% 7-ADD (Biolegend, San Diego, USA). Results were analyzed by Flow Cytometer BD FACSCanto II (BD Bioscience, Heidelberg, Germany).

### Phalloidin staining procedure

Actin staining was performed as described previously [37]. Briefly, for phalloidin staining approx. 20 000 cells/well were seeded onglass cover slides and treated after 7 hours. 48h after plating cells were fixated with PFA 4% and stained with Phalloidin 488 (1:50) and Hoechst (1:10 000). Images were taken by an Axio Observer with the Zen Software (Zeiss, Oberkochen, Germany).

### Fluorescence microscopic imaging

Neurons were treated with novel compounds and their respective solvent controls for 72 h. Afterwards, cells were fixed with 4% formaldehyde. They were stained with β-III-tubulin marker (1:500, Promega, Madison, Wisconsin, USA), astrocytes with GFAP marker (1:500, Dako, Glostrup, Denmark) and the DNA with Hoechst33258 (1:10 000, Life Technologies, Darmstadt, Germany). Pictures were taken by an Axio Observer with the Zen Software (Zeiss, Oberkochen, Germany).

### Cell migration assay

For migration assay 100.000 cells/well were plated into a 12-well-plate and cultured. After 36h a scratch was made across the cell layer with a sterile pipette tip. After imaging the cells (0h) cells were treated with different concentrations of the compounds. Images were taken with Olympus x71 and cell-F-Software (Olympus, Tokyo, Japan) at various times. To quantify the data, the distance between cells was measured using Image J software.

### Statistical analysis

Quantitative data from experiments were obtained as stated in the figure legend. Analysis was performed using unpaired Student’s *t* test if not otherwise stated (MS Excel). Data from all experiments were obtained from at least three independent experiments. The level of significance was set at * = p < 0.05. Error bars represent ± SEM.

### Ethical statements

Studies with human tissue were conducted in compliance with the Helsinki Declaration and approved by the Ethics Committee of the Friedrich-Alexander University of Erlangen-Nuremberg.
